# A Predictive Genetic Signature for Response to Fluoropyrimidine-Based Neoadjuvant Chemoradiation in Clinical Stage II and III Rectal Cancer

**DOI:** 10.3389/fonc.2013.00288

**Published:** 2013-11-25

**Authors:** Jason Chan, Michael T. Kinsella, Joseph E. Willis, Huankai Hu, Harry Reynolds, Conor Delaney, Andrea McCulla, Steve Deharo, Miika Ahdesmäki, Wendy Louise Allen, Patrick G. Johnston, Timothy J. Kinsella

**Affiliations:** ^1^Department of Radiation Oncology, Brown University, Providence, RI, USA; ^2^Department of Radiation Oncology, Memorial Sloan Kettering Cancer Center, New York, NY, USA; ^3^Department of Pathology, Case Medical Center, Cleveland, OH, USA; ^4^Department of Surgery, Case Medical Center, Cleveland, OH, USA; ^5^Almac Diagnostics, Craigavon, UK; ^6^Centre for Cancer Research and Cell Biology, Queen’s University Belfast, Belfast, UK

**Keywords:** rectal neoplasms, ubiquitination, gene array, UBC, EHBP1

## Abstract

**Purpose**: Pre-operative chemoradiation (CRT) is currently the standard of care for patients with clinical stage II and III rectal cancer but only about 45% of patients achieve tumor downstaging and <20% of patients achieve a pathologic complete response. Better methods to stratify patients according to potential neoadjuvant treatment response are needed. We used microarray analysis to identify a genetic signature that correlates with a pathological complete response (pCR) to neoadjuvant CRT. We performed a gene network analysis to identify potential signaling pathways involved in determining response to neoadjuvant treatment.

**Patients and Methods**: We identified 31 T3–4 N0–1 rectal cancer patients who were treated with neoadjuvant fluorouracil-based CRT. Eight patients were identified to have achieved a pCR to treatment while 23 patients did not. mRNA expression was analyzed using cDNA microarrays. The correlation between mRNA expression and pCR from pre-treatment tumor biopsies was determined. Gene network analysis was performed for the genes represented by the predictive signature.

**Results**: A genetic signature represented by expression levels of the three genes EHBP1, STAT1, and GAPDH was found to correlate with a pCR to neoadjuvant treatment. The difference in expression levels between patients who achieved a pCR and those who did not was greatest for EHBP1. Gene network analysis showed that the three genes can be connected by the gene ubiquitin C (UBC).

**Conclusion**: This study identifies a 3-gene signature expressed in pre-treatment tumor biopsies that correlates with a pCR to neoadjuvant CRT in patients with clinical stage II and III rectal cancer. These three genes can be connected by the gene UBC, suggesting that ubiquitination is a molecular mechanism involved in determining response to treatment. Validating this genetic signature in a larger number of patients is proposed.

## Introduction

### Treatment of locally advanced rectal cancer

An estimated 40,340 new cases of rectal cancer will be diagnosed in 2013 (1). Rectal cancer is a highly treatable and often curable disease when localized. Surgery is the mainstay of treatment. The 2-year local recurrence rates after surgery alone for Stage II and III rectal cancers are <10% (2, 3). The 5-year survival rates after surgery alone for T3 and T4 rectal cancers vary from 44 to 60% and from 25 to 30% in patients with lymph node involvement (4).

The addition of postoperative radiation improves local-regional control whilst the use of postoperative chemotherapy is associated with a survival benefit (5). When combined, the use of postoperative radiation together with a fluorouracil-based radiosensitizer significantly improves the results of therapy for rectal carcinoma with a poor prognosis, as compared with postoperative radiation alone (6).

The advent of pre-operative chemoradiation (CRT) represents a new phase in improving rectal cancer treatment allowing for tumor downstaging prior to radical resection. This has been associated with considerable advantages over postoperative approaches (7) including improvement of local disease control (4, 8–14), overall and cancer specific-survival (15), and allowing for sparing of the rectal sphincter in some patients where an abdomino-perineal resection would have been necessary (16, 17). Another important advantage of pre-operative CRT is allowing for pathological assessment of the resected specimen after neoadjuvant treatment, which has been shown to be predictive of treatment outcomes (18–21).

### Predicting response to neoadjuvant chemoradiation

Despite these advantages, the clinical and pathological response to pre-operative CRT is extremely variable between individual patients. Only about 45% of treated patients achieve T stage downstaging (16, 22–24), and <20% of treated patients achieve a pathological complete response (pCR), where no viable tumor cells are found in the resected specimen after neoadjuvant treatment (23, 25, 26). Several predictive clinical factors have been identified including tumor stage, tumor mobility, proportion of rectal circumference involved by tumor, and tumor differentiation (9). Radiation dose and time elapsed from radiation to surgery are also important (4, 13, 27).

Furthermore, such variable individual responses to neoadjuvant treatment raise the question of genetic and epigenetic heterogeneity of rectal tumors and motivate the need to discover predictive biological markers to stratify patients according to potential treatment response. This would ultimately have the benefit of modifying treatment so that, for example, patients with little likelihood of having a therapeutic benefit from pre-operative CRT would be spared the potential morbidity (28). Conversely, patients predicted to have a pCR might be spared the morbidity associated with surgery (29–31).

A recent review of predictive biomarkers for response to neoadjuvant CRT identified six biomarkers with more than five studies in the literature: p53, epidermal growth factor receptor (EGFR), Ki-67, p21, bcl-2/bax, and thymidylate synthase (TS) (32, 33). The predictive value of these biomarkers is mostly derived from immunocytochemistry analyses of pre-operative biopsy material. p53 is the most studied biomarker but conflicting data exists regarding the predictive value of p53 expression and p53 mutations (8, 9, 34, 35). Among these single gene biomarkers, TS currently appears to be the most promising avenue of investigation in predicting response to CRT. TS is the primary target for 5-FU activity and its increased expression has been identified as a poor prognostic and predictive factor in various cancers (36–41). A recent prospective study stratified 37 out of 135 rectal cancer patients into a poor risk group based on genotypes that correlate with increased TS expression and activity (42). However, the data are not consistent throughout the literature as regards the significance of TS expression level as a predictor of response to FU-CRT (32). This is in part due to small study populations and differences in treatment regimens but much of the discrepancy in the literature probably derives from the complex nature of these pathways, which single gene expression assays cannot adequately explain.

One of the most useful recent advances in the ability to investigate biological systems is the advent of cDNA array technology. DNA microarrays contain oligonucleotide or cDNA probes for measuring the expression of thousands of genes in a single hybridization experiment and allow for the potential to discover predictive genetic “signatures” that are represented by numerous genes products (43–46). A recent study discovered a genetic signature of 13 miRNAs that correlated with pCR after neoadjuvant CRT in locally advanced rectal cancer patients (47).

Using cDNA microarrays in this manner, we attempt to identify gene clusters associated with pCR after neoadjuvant CRT in a small group of rectal cancer patients. We obtained formalin-fixed paraffin-embedded specimens taken from pre-treatment biopsies of previously treated patients and identified 8 patients who achieved pCR and 23 patients who did not achieve pCR. We found a genetic signature that was found to be correlated with pCR and was independent of clinical factors. This genetic signature consisted of three genes EHBP1, STAT1, and GAPDH. Network analysis revealed that all three genes can be connected through a single gene Ubiquitin C (UBC), suggesting that ubiquitination may be a molecular mechanism involved in determining response to FU-CRT.

## Materials and Methods

### Patients and tissue specimens

Forty patients treated at the University Hospitals Case Medical Center with clinical Stage II and III rectal cancer who underwent pre-treatment biopsies and post-treatment pathological assessment of tumor response to neoadjuvant CRT were included in this study. The retrospective inclusion criteria were locally advanced T3/T4 N0–1 M0 rectal cancer by endoscopic ultrasound (EUS) in patients who were not previously treated. The exclusion criteria included recurrent colorectal cancer or the presence of a known coagulopathy. Biopsies were taken at the time of initial diagnosis. Patients then received standard CRT. Finally, patients underwent total mesorectal excision. Multiple sections of pre-operative cancer biopsies (average 10 per case) were subjected to laser capture microdissection using an Arcturus Veritas Laser Capture Microdissection Microscope (Applied Biosystems).

### Chemoradiation therapy

Patients received 54–63 Gy at 1.8 Gy daily fractions over 6–7 weeks depending on tumor volume determined by CT and EUS. CT-based 3-D conformal radiation therapy treatment planning was used. 5-FU was administered as a continuous infusion throughout radiation therapy. A dose of 200–250 mg/m^2^/day was administered typically through a surgically placed port.

### Pathology

Post-chemoradiation therapy resection specimens were processed. The resected specimens were fixed in formaldehyde and a minimum of four paraffin blocks were processed for each sample. The tumor response in the surgical resection specimen was graded according to previously published criteria (48). Tumor regression of the primary tumor was measured by laser capture microscopy to compare the amount of tumor versus stromal tissues. Tumor regression scores were assigned as follows: grade 0, no residual tumor; grade 1, rare residual tumor cells; grade 2, fibrosis with residual cancer; and grade 3, dominant residual cancer. pCR was defined by a tumor regression score of 0. Non-pCR was defined by tumor regression scores of 2 and 3. One patient did not receive neoadjuvant radiation therapy and was excluded from analysis. Seven specimens were assigned a tumor regression score of 1 and were excluded from analysis.

### Genetic analysis

mRNA extracted from viable tumor cells derived from formalin-fixed paraffin-embedded tissue were amplified and converted to cDNA using the NuGEN WT-Ovation FFPE RNA amplification V2 kit (NuGEN Technologies, Inc.). The cDNA was fragmented and labeled using the NuGEN Encore kit and hybridized to the Almac Diagnostics Colorectal Cancer DSA^®^ arrays. Each microarray contains cDNA probes for measuring the expression of thousands of genes in a single hybridization experiment. One tumor specimen was identified as an outlier following data quality and integrity assessment and was excluded from the analysis.

### Statistical methods

Following percent present filtering and 50% variance intensity filtering, signature generation starting from 8496 probe sets was conducted. Genetic signatures consisted of predictive probe set signatures of different sizes that were evaluated under cross validation and utilizing forward feature selection for the selection of the optimal signature, following the best practices of the MicroArray Quality Control consortium (49). The performance of each genetic signature was evaluated in two ways: the area under the Receiver Operating Characteristic (ROC) curve (AUC) for response detection and the signature’s independence from clinical covariates. After the highest performing genetic signature was identified, we performed a permutation test by randomly shuffling the response information for the samples. Finally, functional and topological analyses were performed to identify biological entities associated with the gene list represented by our genetic signature using Ingenuity Pathway Analysis^®^ (IPA).

## Results

### Patient characteristics

Two sample groups were identified from a sub-population of 31 rectal cancer patients with varying response to 5-FU and radiotherapy (Table 1). Eight responders were defined as having a tumor regression score of 0 and 23 non-responders defined having a tumor regression score of 2 or 3. At the time of analysis, 16 patients were alive without evidence of disease, 9 were alive with evidence of disease, 4 were alive with unknown disease status, 1 died with evidence of disease, and 1 died with no evidence of disease.

**Table 1:** **Patient characteristics**.

Patient ID	Sex	Patient status	Follow up (months)	Tumor regression regression score
S0740F0024	F	ANED	126	0
S0740F0027	F	ANED	38	0
S0740F0028	M	ANED	13	0
S0740F0032	M	DNED	55	0
S0740F0034	F	ANED	56	0
S0740F0040	M	AWD	5	0
S0740F0001	M	ANED	52	0
S0740F0002	M	ANED	27	0
S0740F0017	M	AWD	6	2
S0740F0020	M	AWD	24	2
S0740F0030	M	ANED	15	2
S0740F0036	M	AWD	99	2
S0740F0038	F	DWD	14	2
S0740F0025	M	ANED	48	3
S0740F0026	F	ANED	53	3
S0740F0029	M	AUNK	14	3
S0740F0031	M	AWD	21	3
S0740F0033	F	AWD	14	3
S0740F0035	M	AUNK	3.5	3
S0740F0037	M	ANED	14.5	3
S0740F0039	M	AUNK	29	3
S0740F0004	M	AUNK	17	3
S0740F0005	M	AWD	54	3
S0740F0006	M	ANED	48	3
S0740F0007	M	ANED	30	3
S0740F0008	M	ANED	46	3
S0740F0009	M	AWD	27	3
S0740F0010	M	ANED	47	3
S0740F0011	F	AWD	7	3
S0740F0012	F	ANED	48	3
S0740F0016	M	ANED	48	3

Patient status: ANED, alive, no evidence of disease; AWD, alive with disease; DWD, dead with disease; DNED, dead, no evidence of disease; AUNK, alive disease unknown. Tumor regression score: 0 = no residual tumor; 2 = fibrosis with residual cancer; 3 = dominant residual cancer, no regression.

### Genetic signature generation

Gene expression signatures were generated in rectal cancer patients treated with neoadjuvant CRT. A 4-probe set signature was discovered to be the highest performing genetic signature (Table 2). Each probe set consists of genetic probes with a single genetic target. One probe set targets an antisense transcript and therefore does not provide information about gene function. The other three genes represented by this genetic signature include EHBP1, STAT1, and GAPDH.

**Table 2:** **List of genes that is represented by the genetic signature**.

ProbeSet ID	Gene symbol	Gene title	Entrez gene
ADXCRAD_CX760189_at	EHBP1	EH domain binding protein 1	23301
ADXCRAG_AL831944_at	Antisense	Antisense	Antisense
AFFX-HUMISGF3A/M97935_MA_at	STAT1	signal transducer and activator of transcription 1, 91 kDa	6772
AFFX-HUMGAPDH/M33197_5_at	GAPDH	Glyceraldehyde-3-phosphate dehydrogenase	2597

The AdXCRAG_AL831944 probe set is an antisense transcript and does not represent gene function. The genetic signature, therefore, represents the three genes: EHBP1, STAT1, and GAPDH.

**Figure 1:**
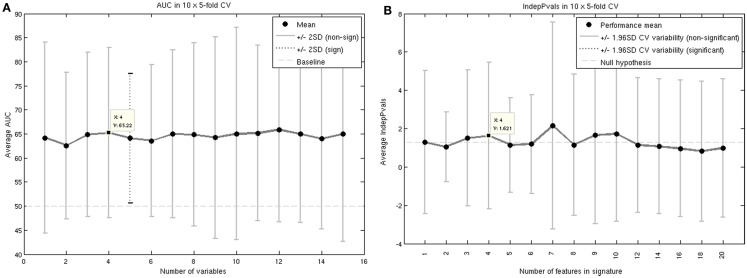
**AUC (A) and independence to clinical covariates p-values (B) for the highest performing genetic signature**.

Repeated testing of patient samples showed that the average area under ROC curves (AUC) was 65%. To test whether this genetic signature was an independent predictive factor, we identified a list of clinical factors to rule out intercorrelations (Table 3). The clinical covariate independence *p*-values were on average below 0.05 or above 1.3 on the log scale (Figure 1).

**Table 3:** **List of clinical covariates used in testing for independence of genetic signature prediction**.

Clinical covariates
1. Follow up length
2. Date of last follow up
3. Gender
4. Patient status
5. Tumor regression score
6. Response
7. Ethnicity

**Figure 2:**
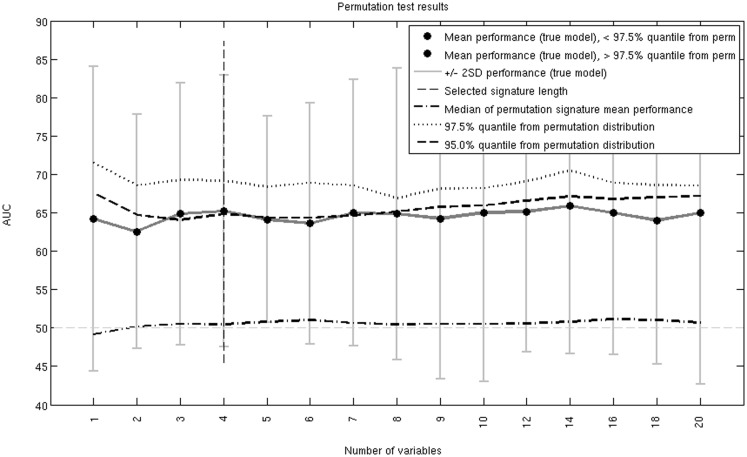
**Permutation test AUC results over all considered signature lengths**. The 4-probe set genetic signature is indicated with the vertical dashed line. The true AUC curve is just above the 95% but below the 97.5% quantile.

This signature was further studied in a permutation analysis. A null hypothesis AUC distribution was produced by randomly shuffling the patient response information. The median, 95, and 97.5% quantiles are shown in Figure 2. The 4-probe set genetic signature on average generates an AUC above the 95% quantile but below the 97.5% quantile. Therefore, the selected signature does not pass permutation testing at the stringent 97.5% quantile or *p*-value of 0.05 threshold, but this may, in part, be due to the small sample size.

### Functional analysis of the genetic signature

**Figure 3:**
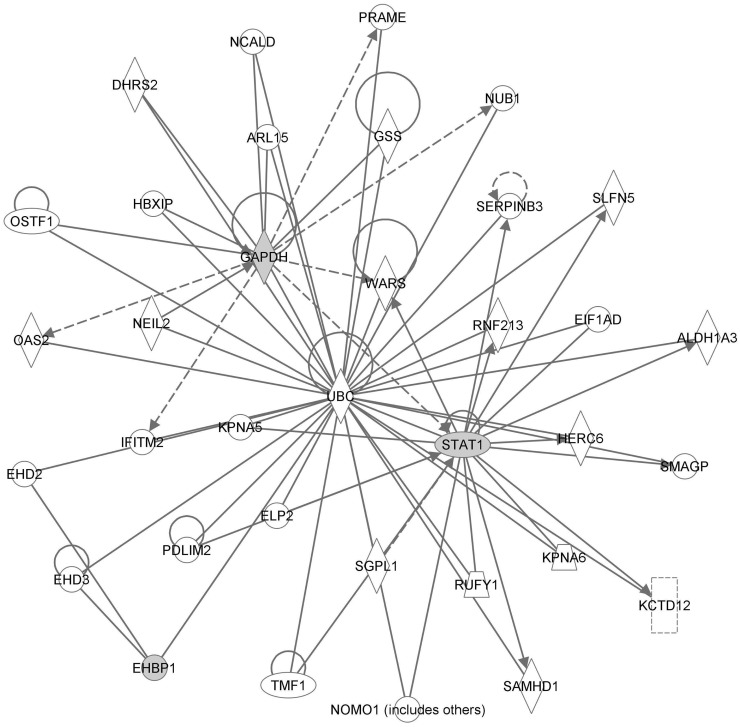
**Network analysis results**. Genes present in the genetic signature are colored gray.

Network analysis revealed that all three genes represented by the genetic signature can be connected through a single gene UBC (Figure 3). Furthermore, the probe set for EHBP1 was identified as the strongest differentiator between the responders and non-responders.

## Discussion

This study identifies a genetic signature that is predictive of pathologic complete response (pCR) to neoadjuvant CRT and found to be independent from clinical factors. The genetic signature was represented by the three genes: EHBP1, STAT1, and GAPDH. EHBP1 expression level was found to be the greatest differentiator between patients that achieved pCR and patients that did not. Functional analysis revealed good connectivity between the genes as they can all be connected through the gene UBC.

### pCR predicts treatment outcomes

The correlation between this novel genetic signature and pCR is significant because pCR is a predictor of treatment outcomes. Local control rates are improved in patients who achieve pCR (23). Additionally, the 5-year disease-free survival is about 83% for patients that achieve pCR and about 66% for those that do not (19, 48). Validating this genetic signature in a larger study population will be needed to assess whether this genetic signature is predictive of treatment outcomes in addition to pCR.

### EHBP1, STAT1, GAPDH, and ubiquitination

EHBP1 is a gene that is thought to be involved with endocytic trafficking by mediating actin reorganization (50). An intron of EHBP1 is known to bear two SNPs that are associated with prostate cancer (51), but otherwise this gene has not previously been linked with cancer. Whether these SNPs affect gene expression or other downstream signaling effects are also currently unknown.

STAT1 is transcription factor complex that is involved in pro-apoptotic and anti-proliferative signaling pathways (52), and has been implicated in numerous cancers including colorectal cancer (53). Specific ligand-receptor tyrosine kinase binding induces tyrosine phosphorylation by JAK1 and JAK2, which recruit STAT1 molecules to form STAT1 dimers. STAT1 dimers translocate to the nucleus and function as part of a transcription factor complex. Increased expression of STAT1 has been reported to be a favorable prognostic factor in colorectal cancer (54, 55).

GAPDH catalyzes the sixth step in glycolysis and has been implicated in promoting apoptosis (56). GAPDH has been reported to be overexpressed in colorectal cancer (57), but has not been studied as a prognostic or predictive factor.

Ubiquitin C is a small regulatory protein that labels proteins to be transported and destroyed in proteasomes (58), and its role in colorectal carcinogenesis and colorectal cancer prognosis was recently reviewed (59). In most sporadic colorectal cancers, mutations in APC prevent the ubiquitination of β-catenin, which is a transcription factor involved in cell proliferation. The downstream effects are that β-catenin is not recognized by the proteasome and excess β-catenin will translocate into the nucleus.

## Conclusion

This study identifies a 3-gene signature that correlates with a pCR to neoadjuvant CRT in patients with clinical stage II and III rectal cancer. These three genes can be connected by the gene UBC, suggesting that ubiquitination is a molecular mechanism involved in determining response to neoadjuvant CRT.

## Conflict of Interest Statement

The authors declare that the research was conducted in the absence of any commercial or financial relationships that could be construed as a potential conflict of interest.
